# Hospital Provider’s Perspectives on MOUD Initiation and Continuation After Inpatient Discharge

**DOI:** 10.1007/s11606-024-09008-x

**Published:** 2024-11-25

**Authors:** Riley Shearer, Honora Englander, Hildi Hagedorn, Adetayo Fawole, JoAn Laes, Hope Titus, Alisa Patten, Emily Oot, Noa Appleton, Amy Fitzpatrick, Roxanne Kibben, Jasmine Fernando, Jennifer McNeely, Dave Gustafson, Noa Krawczyk, Zoe Weinstein, Paulette Baukol, Udi Ghitza, Tracy Siegler, Gavin Bart, Angela Bazzi

**Affiliations:** 1 University of Minnesota School of Public Health, Minneapolis, MN USA; 2 Oregon Health and Science University, Portland, OR USA; 3 Minneapolis Veterans Affairs Health Care System, Minneapolis, MN USA; 4 New York University Grossman School of Medicine, New York, NY USA; 5 Hennepin Healthcare Research Institute, Minneapolis, MN USA; 6 Boston University, Boston, MA USA; 7 Boston Medical Center and the Boston University Chobanian & Avedisian School of Medicine, Boston, MA USA; 8 University of Wisconsin, Madison, WI USA; 9 National Institute On Drug Abuse, North Bethesda, MD USA; 10 Hennepin Healthcare, Minneapolis, MN USA; 11 Herbert Wertheim School of Public Health, University of California San Diego, La Jolla, CA USA

**Keywords:** Medications for opioid use disorder, Hospital addiction medicine, Care transitions, Implementation

## Abstract

**Background:**

Individuals with opioid use disorder have high rates of hospital admissions, which represent a critical opportunity to engage patients and initiate medications for opioid use disorder (MOUD). However, few patients receive MOUD and, even if MOUD is initiated in the hospital, patients may encounter barriers to continuing MOUD in the community.

**Objective:**

Describe hospital providers’ experiences and perspectives to inform initiatives and policies that support hospital-based MOUD initiation and continuation in community treatment programs.

**Design:**

As part of a broader implementation study focused on inpatient MOUD (NCT#04921787), we conducted semi-structured interviews with hospital providers.

**Participants:**

Fifty-seven hospital providers from 12 community hospitals.

**Approach:**

Thematic analysis examined an emergent topic on challenges transitioning patients to outpatient MOUD treatment and related impacts on MOUD initiation by inpatient providers.

**Key Results:**

Participants described structural barriers to transitioning hospitalized patients to continuing outpatient MOUD including (a) limited outpatient buprenorphine prescriber availability, (b) the siloed nature of addiction treatment, and (c) long wait times. As a result of observing these structural barriers, participants experienced a sense of futility that deterred them from initiating MOUD. Participants proposed strategies that could better support these patient transitions, including developing partnerships between hospitals and outpatient addiction treatment and supporting in-reach services from community providers.

**Conclusions:**

We identified concerns about inadequate and inaccessible community-based care and transition pathways that discouraged hospital providers from prescribing MOUD. As hospital-based opioid treatment models continue to expand, programmatic and policy strategies to support inpatient transitions to outpatient addiction treatment are needed.

**NCT Trial Number:**

04921787.

**Supplementary Information:**

The online version contains supplementary material available at 10.1007/s11606-024-09008-x.

## INTRODUCTION

Opioid-related hospitalizations continue to grow across the United States^1^ and are increasingly recognized as opportunities to engage patients in addiction treatment and initiate medications for opioid use disorder (MOUD).^2,3^ Numerous studies show that both buprenorphine and methadone can be successfully initiated in the hospital setting.^4,5^ Furthermore, receipt of MOUD in the hospital is associated with increased rates of ongoing community treatment following discharge from the hospital.^6–8^ However, widespread implementation of hospital-based opioid treatment remains limited, restricting patient access to MOUD in the inpatient setting and missing opportunities to engage and connect patients to longer-term treatment.^9^ In the community, buprenorphine can be prescribed broadly in general and specialty health settings by any DEA-registered provider. Methadone can only be dispensed in more tightly regulated opioid treatment programs.

Previous studies among hospital providers have identified barriers related to limited community substance use treatment capacity and challenges transitioning patients to outpatient treatment.^10–12^ How hospital providers’ willingness to adopt MOUD treatment is impacted by these barriers transitioning patients to continuing community treatment has not been widely documented. Additionally, a deeper understanding of barriers to transitioning hospital patients to continuing opioid use disorder treatment could help inform the development and implementation of strategies to support this transition.^13^ Strategies to alleviate these barriers could help increase access to MOUD in both inpatient and outpatient settings. As part of an effort to inform the widespread implementation of a hospital-based opioid treatment (HBOT) model, we conducted qualitative interviews with hospital care team members across four states. These interviews explored existing approaches for treating patients with OUD, perceptions of community treatment availability, and practical suggestions for improving continuity of care.

## METHODS

### Study Design and Sample

For this study, we drew from baseline qualitative data collected within an ongoing implementation trial (NCT#04921787) exploring community hospitals’ readiness to increase MOUD prescribing.^14^ To be eligible for the trial, community hospitals had to identify a champion to support implementation and could not already have a hospital-based opioid treatment program or addiction consultation service. From the 24 hospitals in the study, 12 were randomized to the “high-intensity” arm which included baseline qualitative interviews. Each hospitals’ champion was interviewed as well as three to five individuals with in-depth knowledge of hospital practices for treating OUD and barriers to implementing MOUD prescribing. Purposive^15^ and snowball samplings^16^ were used to identify approximately three to five individuals with diverse roles at each hospital. Some interviewees had administrative duties, but all had experience in patient facing roles (e.g., physicians, physician assistants [PAs], nurse practitioners [NPs], nurses, social workers, and case managers).^14^ All participants provided verbal informed consent and were offered $50 compensation for their time. These study procedures were reviewed and approved by the Advarra, Inc., institutional review board (00047868).

### Data Collection

Between November 2021 and March 2022, trained study personnel who had not previously interacted with participants conducted semi-structured interviews with participants. Interviews including open-ended questions and detailed probes designed to explore perspectives on potential barriers to increase MOUD prescribing in the inpatient setting (Appendix A). In addition to broad questions about barriers to increasing MOUD prescribing (e.g., “What kinds of issues or complications could arise with efforts to increase access to MOUD within the inpatient setting?”), the interview guide included questions and probes about the communication and relationships between inpatient and outpatient settings (e.g., “How do you collaborate across inpatient and outpatient settings?” and “Does your hospital currently provide any MOUD through a referral to an outpatient clinic?” and “How available are providers waivered to prescribe buprenorphine?”). Interviews were one-on-one, conducted virtually via Zoom videoconferencing or by phone, and lasted approximately 45–60 min. Audio transcripts were recorded and professionally transcribed and de-identified following a structured protocol.^17^

### Data Analysis

We reviewed transcripts to identify emergent themes. Using a collaborative codebook development process,^18,19^ initial codes, sub-codes, and definitions were based on key interview questions, interviewers’ notes, and relevant literature. After six rounds of testing and refining the codebook, analysts used NVivo (QSR International Pty Ltd., release 1.7.1, 2022) to apply final codes to each transcript. In ongoing team meetings to monitor coding accuracy, we noted that participants frequently discussed challenges transitioning patients to the outpatient setting post-discharge, which emerged as a barrier to MOUD initiation in the inpatient setting. To further explore participants’ experiences transitioning patients to continuing MOUD treatment, perspectives on outpatient OUD treatment availability, and related impacts on inpatient care, we conducted an in-depth thematic analysis qualitative interview data initially coded with “discharge with MOUD” and “outpatient and community treatment.” After carefully rereviewing this data, the lead author identified initial themes (with supporting quotations) that we then refined through discussions with interviewers and investigators who have research and clinical experience treating OUD in a hospital setting.

## RESULTS

### Overview of Sample and Key Findings

**Figure 1:**
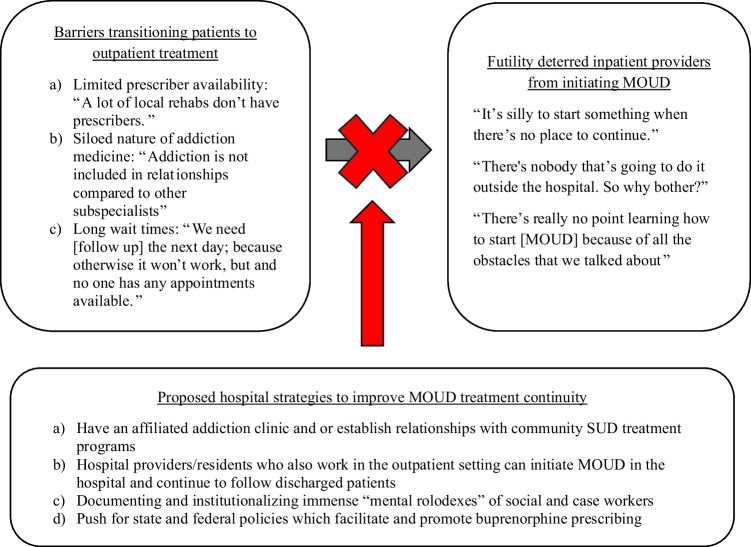
Impact of barriers transitioning patients to continued care on perceived futility of initiating MOUD and proposed strategies

Across all 12 community hospitals, 57 hospital providers were interviewed. Participants included physicians (*n* = 30), pharmacists (*n* = 7), nurses (*n* = 6), NPs and PAs (*n* = 3), and additional professionals engaged in the care of patients with substance use disorders (e.g., social workers, peer recovery specialists, addiction counselors, substance use navigators; *n* = 11). From qualitative interviews, we identified several interrelated themes: First, participants described barriers to transitioning hospitalized patients to outpatient providers. Second, participants described how these barriers resulted in hospital providers feeling discouraged from initiating MOUD. Finally, participants also provided suggestions for strategies that could help overcome these challenges and improve transitions from inpatient to outpatient addiction treatment (Fig. 1).

#### Barriers to Transitioning Hospitalized Patients to Outpatient MOUD Treatment

Participants felt that challenges transitioning patients between hospital and outpatient treatment were a key barrier limiting MOUD initiation during hospitalizations: “Our biggest challenge when we start patients on medication is where do we send them for follow up? In the ten years I’ve been here, that’s always been the main problem” (social worker 1). Participants described specific challenges that they viewed as being outside of hospitals’ control, including *(a) limited buprenorphine prescriber availability, (b) siloed nature of addiction treatment, and (c) long wait times.*

##### Limited Buprenorphine Prescriber Availability

Some participants felt a paucity of community providers who were trained and willing to prescribe buprenorphine contributed to outpatient treatment shortages and impeded discharge planning for patients receiving buprenorphine. Inpatient providers’ awareness of outpatient treatment availability was limited to the few hospitalized patients they treated who were already receiving buprenorphine. For example, one physician reflected that they only knew of patients receiving buprenorphine from pain management specialists, but not primary care or addiction medicine doctors. Another hospital physician acknowledged there may be more community providers trained and willing to prescribe buprenorphine than they were aware of. But without formal discharge planning and treatment transition processes in place, they did not know who would prescribe MOUD: “There’s many more primary care providers in our community, but our professional lives don’t intersect; I’m a hospital doc, and these guys don’t come to the hospital” (physician 1). In addition to primary care settings, even some specialty substance use treatment facilities often refused to continue MOUD, as one physician explained, “A lot of local rehabs don’t have prescribers” (physician 2). As a result, there was no licensed provider who could prescribe buprenorphine for patients at these substance use treatment facilities.

##### Siloed Nature of Addiction Treatment

Participants noted how the siloed nature of addiction treatment services impeded transitions between inpatient and outpatient treatment. Some healthcare systems had no formal outpatient addiction medicine services, and so patients at these community hospitals had to seek ongoing substance use disorder treatment in a separate system. In other instances, the siloing of addiction medicine was reinforced by the physical and organizational distance between addiction clinics and other healthcare facilities, which also limited relationships between inpatient and outpatient providers: “We have a beautiful building where all of our specialists sit along with primary care. But not addiction, addiction is across the river [and] not included in the relationships compared to other subspecialists” (physician 3).

Some hospitalized patients with OUD required continuing care in a post-acute medical care facility or substance use treatment facility. Hospital providers struggled to find facilities to provide both post-discharge physical medical care and MOUD. As a result, providers were forced to prioritize treating either OUD or other complex medical conditions during a patient’s hospitalization. As one NP described, “The problem that I have is if we’re going to discharge somebody to a skilled nursing facility then we can’t prescribe Methadone. So then we’re stuck: how are we gonna keep them out of withdrawal, if when they leave the hospital, they need this rehab?” (NP 1). Without established partnerships with opioid treatment programs, post-acute medical care facilities are unable to dispense methadone.

##### Long Wait Times

Hospital staff encountered excessively long wait times when trying to connect patients with outpatient MOUD treatment (e.g., 1 to 2 weeks following hospital discharge). Without timely follow-up, participants worried that patients would not continue treatment and return to substance use, as one NP explained, “Whatever we did in the hospital does not translate to maintenance therapy [or] actual control of the disease process” (NP 2). This feeling was expanded upon by a physician who felt that the success of hospital-based MOUD initiation was predicated on access to immediate outpatient follow-up: “We need [follow up] the next day; that’s the agreement we need, because otherwise it won’t work…[but] everyone’s busy and no one has any appointments available, and it is what it is” (physician 4). Although participants acknowledged that providing appropriate treatment in the hospital could improve patient outcomes, some expressed concern that providing MOUD would increase patients’ length of stay. “We’ll have a patient medically stable, started on [MOUD], and then we [can’t] discharge because there’s no place to [send them] in the community [without] a seven-day wait period” (NP 1). Without timely outpatient treatment options available, initiating MOUD and reducing length of stay were viewed as competing priorities.

#### Consequences of Structural Barriers to Transitioning Patients to Outpatient MOUD Treatment

These barriers transitioning hospitalized patients to outpatient MOUD treatment discouraged providers and resulted in feelings that MOUD initiation in the inpatient setting was futile discouragement among inpatient providers.

##### Futility Deterred Inpatient Providers from Initiating MOUD

The aforementioned challenges to MOUD access in outpatient settings contributed to general uncertainty and discouraged providers from initiating inpatient treatment: “I think if we had someplace for these patients to continue it, we’d be more inclined to [initiate] it. To me, it’s silly to start something when there’s no place…to continue” (physician 5). Among providers who were not yet comfortable prescribing MOUD, perceived barriers also discouraged them from investing time in learning how to initiate treatment with MOUD. As one NP expressed, “[Prescribing] doesn’t happen because we don’t know how to do it, and there’s nobody that’s going to do it outside the hospital. So why bother?” (NP 1). Another physician tied the sense of futility created by the barriers transitioning patients to continuing MOUD treatment to not learning how to prescribe MOUD or increasing efforts to improve MOUD prescribing:

There’s really no point learning how to start [MOUD] because of all the obstacles that we talked about…Why talk to [patients] about it in the hospital [and] why spend 8 or 20 hours learning how to do that? […] It’s a ‘catch-22.’ You’ve got to have enough infrastructure for people to feel like it’s worth their time (physician 6).

Feelings of futility were compounded by some providers’ view that the inpatient setting was inappropriate to manage chronic conditions. For example, a physician explained that although they would manage acute withdrawal symptoms it was the responsibility of outpatient providers to initiate and manage MOUD and other chronic medical treatment: “We would definitely treat acute issues [and] acute withdrawal symptoms. That’s [a] high priority; a crisis for [the patients]. And then we would transition them [elsewhere] because we don’t focus on anything beyond acute [care]” (physician 7).

Perceived futility frustrated providers who felt they could not help patients continue MOUD due to structural barriers transitioning to outpatient treatment. One physician described MOUD prescribing in hospitals as “temporary” and not accomplishing “anything actually constructive” (physician 8) without adequate outpatient options. Similarly, another physician described feeling the care they offered was inadequate, commenting that “ignoring their OUD while treating their infection feels a little negligent to me” (physician 6). Another physician described feeling demoralized by not adequately treating patients with OUD: “You run into this tendency to groan when someone [with OUD] comes in…because we’re not doing a good job, and we know we don’t have a lot to offer, so that’s an example of the morale piece of it” (physician 8).

#### Proposed Hospital Strategies to Improve MOUD Treatment Continuity

In addition to recognizing barriers, some participants also described strategies to overcome barriers and facilitate transition to ongoing community treatment with MOUD. A physician at one hospital emphasized the benefit of having an affiliated addiction medicine clinic: “We have an outpatient clinical practice we can send patients to for follow up; that makes a big difference for us and maybe gets over some of those perceived barriers” (physician 9). This participant highlighted how an established relationship with an outpatient clinic reduced the siloed nature of addiction medicine and made it easier to know where to transition patients with care. Another hospital, which did not have affiliated addiction medicine services, instead developed collaborative relationships with local SUD treatment programs. Outpatient treatment programs offered in-reach services, meeting with patients while they were still in the hospital, to facilitate warm handoffs. At both hospitals, participants suggested that relationships with outpatient providers and established processes for transitioning patients to community treatment increased hospital providers’ willingness to initiate MOUD in the hospital.

Other participants described staffing arrangements that enabled individual providers to initiate MOUD in the hospital and continue it in outpatient settings. While most providers worked full-time as hospitalists, a few worked part-time in outpatient clinics where they could see patients who they, or their colleagues, started on MOUD during a recent hospital stay. This practice model was also described at teaching hospitals where medical residents frequently worked both in the hospital and outpatient settings. Having colleagues who also worked in outpatient settings increased inpatient providers’ confidence that MOUD would be continued following hospital discharge.

Participants highlighted that a “lack of knowledge of where we can send patients is the biggest barrier” (physician 10) and emphasized the importance of knowledgeable social workers and case managers in successfully transition patients to community treatment. At multiple hospitals, social workers “sheer [number of] connections” (pharmacist 1), “wealth of resources”, and “passion for carrying these patients through the discharge process into the community” (physician 11) were critical to enabling treatment continuity following hospital discharge. However, the social workers and case managers with extensive “mental rolodex[es]” (addiction counselor 1) already carried an “enormous patient load” (pharmacist 1). Participants stressed the importance of documenting and institutionalizing these connections to increase their ability to transition patients to ongoing treatment without overly relying on any single person.

## DISCUSSION

In this qualitative study, hospital staff described the substance use treatment system as challenging to navigate with an inadequate capacity to provide necessary MOUD for patients leaving the inpatient setting. Importantly, we found that perceived barriers to transitioning hospitalized patients to continuing care discouraged providers from initiating MOUD treatment during hospitalization in the first place. Participants identified numerous critical gaps in community treatment infrastructure and the referral pathways including a lack of partnerships or affiliation between hospitals and community clinics or programs, long wait times for follow-up care, and few buprenorphine prescribers. Discouraged by what was perceived to be a fractured, inaccessible, and sometimes non-existent outpatient treatment system, hospital providers were reluctant to adopt MOUD prescribing. To encourage adoption of HBOT models, hospitals must provide support for transitioning patients to continuing treatment.

For patients initiated on MOUD in the hospital, transition to ongoing community treatment is critical for ensuring success and should be minimally disruptive.^20^ However, national studies of hospitals show that transitions to community care are rarely implemented as part of hospital-based initiatives to address substance use disorders.^21^ As part of a broader study on the implementation of HBOT models, these findings contextualize how barriers transitioning patients to continuing treatment can discourage inpatient providers from initiating MOUD treatment and offer suggestions to improve treatment continuity. For example, participants identified barriers directly impacting patient care, including long wait times to outpatient appointments. Increased wait times are especially problematic because patients who are unable to access a same- or next-day appointment are less likely to be retained in treatment with MOUD.^22^ To reduce wait times and improve patient retention, hospitals should develop partnerships with community treatment providers and invest in transition strategies that support transitioning patients to community treatment.

Beyond improving the patient experience, our findings suggest that investments in strategies to better facilitate the transition from inpatient to outpatient care may in turn increase hospital provider adoption of treatment with MOUD. Indeed, the transitions literature for facilitating transition from acute care settings to outpatient care is steadily growing.^13,23^ One strategy that has been successfully implemented to address some of these challenges is low-barrier bridge clinics, or clinics which accept walk-in appointments, so that there is always an option for discharging patients treated with buprenorphine (and sometimes methadone) while they are being transitioned to long-term community providers.^24^ Similarly, hospitals can develop partnerships with opioid treatment programs to facilitate hospital-based methadone initiation and seamless patient transition at discharge.^4^ In line with recent quantitative findings,^25,26^ participants in this study described challenges identifying post-acute medical care and substance use treatment facilities which would continue treatment with MOUD. Federal regulations complicate the ability of patients to continue methadone in post-acute care medical facilities. As highlighted by participants, developing partnerships with community treatment programs and post-acute medical care facilities can help overcome these barriers. For example, a recent pilot program demonstrated that partnerships between a hospital, opioid treatment programs, and skilled nursing facilities could help integrate MOUD into skilled nursing facilities and supported patient engagement throughout multiple care transitions.^27^ Finally, even in resource-limited settings lacking formal partnerships with outpatient treatment programs, improving bi-directional communication and care coordination may help overcome barriers such as the siloed nature of addiction medicine or the perceived lack buprenorphine prescribers available.^11,28^

In this study, we found that in some instances a lack of knowledge, rather than actual absence of viable community treatment options, was the biggest barrier. It is important that hospitals have systematized processes in place for collecting and documenting community treatment and resources available to patients following hospital discharge. Furthermore, providing staff with dedicated time and training to develop and maintain relationships with community treatment providers may encourage the expansion of MOUD initiation in the inpatient setting.

Beyond formal hospital programs, members of the inpatient care team can also take specific steps to alleviate the barriers identified in this study and support transition to ongoing treatment. For example, when patients face longer wait times, providers can offer bridge prescriptions of buprenorphine or dispense 72 h of methadone at the time of hospital discharge so that patients maintain access to MOUD while waiting for an outpatient appointment. In addition to being knowledgeable of local resources and programs that support patient transitions, it is also important that care coordinators advocate appropriate timing for discharge so patients can receive same- or next-day treatment appointments. Each aspect of the care pathway including hospital-based MOUD initiation, care transitions, and continuing outpatient treatment should support patients’ goals and minimize burdens on patients to increase MOUD retention and encourage wider adoption of HBOT by hospital providers.

In addition to hospital- and individual-level practices, policy changes that improve MOUD access may reduce barriers transitioning patients to community treatment and further increase hospital-based initiation of MOUD. The requirement to have an X-waiver to prescribe buprenorphine was removed since these interviews were conducted. However, the X-waiver removal may not increase the number of community providers prescribing buprenorphine.^29,30^ Findings from this paper emphasize the importance of relationships with community partners and clear treatment pathways so hospital providers are confident patients will be able to continue MOUD treatment. State and federal policies should facilitate treatment transitions from the hospital to the community. For example, telehealth to provide MOUD increased, during the COVID-19 pandemic, and was feasible for both patients and providers.^31–36^ To support increased MOUD access, hospitals should invest in telehealth systems and provide resources, such as cellphones, to vulnerable patients to support transition to community treatment.^37,38^ States can and should adopt policies which reduce barriers to buprenorphine, fund interdisciplinary addiction care teams, and encourage more providers to offer MOUD.^39^ Incentives for providers may also increase community availability of buprenorphine following hospital discharge.^40^ Changes to methadone restrictions could also increase access to MOUD in both hospital and outpatient settings. Although methadone can legally be dispensed in hospitals to treat opioid withdrawal, continued misunderstanding of the law leads to hesitance to offer methadone in hospitals.^41^ Furthermore, allowing office-based prescribing and pharmacy dispensing of methadone could help patients continue to access treatment following hospital discharge.^42^

This study has several limitations. First, this targeted thematic analysis drew from a larger qualitative study designed to describe the facilitators and barriers to implementing hospital-based opioid treatment. Although that parent study included questions about community treatment, transition strategies, and opportunities for improvement, it was not designed to elucidate every aspect of the transition between inpatient and outpatient settings, and we may have missed opportunities to systematically probe about each of the findings described here. Instead, we undertook this thematic analysis because preliminary review of our qualitative data revealed that deficiencies in community treatment options and transition processes emerged as important concerns of our participants. Second, sampled providers were from community hospitals in four geographic regions of the United States, and our results may not be generalizable across all hospitals or regions with different community treatment services or post-discharge patient transfer practices. Additionally, as part of the parent study, participants were disproportionately physicians and recruited for their interest in improving treatment of OUD within their hospitals, likely representing a group with different expertise and perspectives from other hospital providers. Finally, our study did not include community providers who likely have important insight of hospital to community treatment transition processes.

In this qualitative study, we identified barriers to transitioning patients continuing community treatment with MOUD, including limited prescriber capacity, long wait times, the siloed nature of addiction medicine clinics, and restrictive policies. These perceived gaps in community treatment infrastructure and barriers to transitioning hospitalized patients to ongoing care deterred hospital providers from prescribing MOUD. In addition to encouraging providers to adopt treatment with MOUD, hospitals must invest in partnerships with community treatment providers and transition strategies which support patient continuity in treatment. In addition to improving patient care, alleviating barriers to transitioning patients with ongoing treatment may encourage more hospital providers to adopt an HBOT model of care.

## Supplementary Information

Below is the link to the electronic supplementary material.

Supplementary file1 (PDF 36 KB)

## Data Availability

De-identified data from this study may be available from the corresponding author upon reasonable request.
