# Supplementary material for: Hospital Provider’s Perspectives on MOUD Initiation and Continuation After Inpatient Discharge
Source: J Gen Intern Med. 2024 Nov 25;40(12):2926–33. doi: 10.1007/s11606-024-09008-x (PMC12463795; doi:10.1007/s11606-024-09008-x)
Supplement: Supplementary file 1 — Supplementary file1 (PDF 36 KB) [file 11606_2024_9008_MOESM1_ESM.pdf]

Appendix A. CTN 98B Baseline (Pre-Implementation) Qualitative Interview Guide

1. To get started, could you please describe your role within your hospital?

2. What kind of work, if any, have you done with patients with opioid use disorder, or OUD, especially in the hospital setting?

3. How do you think the opioid crisis has impacted your hospital?

4. How does your hospital currently treat patients with OUD?

   a. What hospitals policies are you aware of that govern how people who use drugs are treated?

5. Walk me through how an inpatient with an OUD diagnosis would be treated.

6. Does your hospital currently provide any medications for OUD (or MOUD), either on-site or through contract services?

   [If not offering medications → SKIP TO QUESTION 7]

   a. How consistently is MOUD provided to patients in these settings? How are patients identified for MOUD?
   b. What requirements must patients meet to receive MOUD?
   c. How are patients on MOUD retained in care? Who is involved in this?
   d. In your opinion, how available are providers waivered to prescribe buprenorphine?
   e. Is MOUD prescribed for detox/tapering, or for long-term maintenance?
   f. Under what circumstances would a patient no longer be eligible for MOUD?

7. What plans, if any, does your hospital (or clinic/unit) have to increase access to MOUD in the inpatient setting?

8. In your opinion, how well do medications for the treatment of OUD fit within the existing work processes and practices in your hospital?

9. Based on your experience working here, what kinds of issues or complications could arise with efforts to increase access to MOUD within the inpatient setting?

10. What are the biggest struggles that patients with OUD experience in your hospital?

    a. What are the biggest struggles that staff have when working with patients with OUD?
    b. What kind of information or "proof" about MOUD do you think would help get more staff on board with increasing access to MOUD in the inpatient setting?
    c. What specific resources would help expand access to MOUD within the inpatient setting?

    d. What do you view as the primary goal(s) of MOUD for patients with OUD?

11. Please tell me the roles of the people who would need to be involved in helping to improve access to MOUD in the hospital.

a. Who would be the most influential types of people to get on board with expanding access to medication treatment?
b. As far as you know, how supportive has hospital leadership been of efforts to expand access to MOUD?

12. How do you work with colleagues to care for patients?

13. How do you collaborate across inpatient and outpatient settings?

    a. Does your hospital currently provide any MOUD through a referral to an outpatient clinic?

14. In general, are you aware of any other efforts to expand access to MOUD in the hospital?

15. Can you describe any other initiatives or quality improvement efforts that have been undertaken successfully at your hospital?

16. Based on everything I've asked you about, who else do you think we should speak with to learn about these topics at your hospital? This could include individuals within the inpatient or outpatient settings.

17. OK, reflecting on our discussion, is there anything else we haven't talked about today that you think I should know?
